# What Else? The Basics and Beyond for Effective Consultations with Youth with Special Healthcare Needs

**DOI:** 10.3390/healthcare5040069

**Published:** 2017-10-01

**Authors:** April S. Elliott, Monique C. Jericho

**Affiliations:** 1 Department of Paediatrics, University of Calgary, Calgary, AB T2N 1N4, Canada; 2 Department of Psychiatry, University of Calgary, Calgary, AB T2N 1N4, Canada; monique.jericho@ahs.ca

**Keywords:** adolescent, chronic illness, consultation, coaching

## Abstract

Youth with special healthcare needs (YSHCN) require medical support for disease management and equally require that providers be responsive to their ever-changing and sometimes unique psychosocial and developmental needs. This paper reviews the fundamentals of adolescent consultation reminding the reader that YSHCN are, after all, still youth with the same basic needs as their healthy peers. Beyond the basics, consultations with this population are characterized by complexities which are best managed by providers who can nimbly adjust their clinical stance. In non-urgent clinical scenarios, clinicians can adopt a coaching stance which we introduce and expand upon in this paper. Characterized by the five elements of non-judgment, curiosity, empathy, openness, and flexibility, the coaching stance can be adopted without specific training. We demonstrate its application using TGROW (Topic, Goal, Reality, Options and Wrap Up), a coaching framework that holds promise for use in clinical settings. Consultants may consider incorporating the coaching stance and TGROW into their practice repertoire, as both may be particularly helpful when consulting with adolescents with chronic illness.

## 1. Introduction

The literature shows that approximately 12–18% of adolescents currently live with a chronic illness, and that many healthcare providers (HCP) will encounter youth with special health care needs (YSHCN) in their practice [1,2,3]. YSHCN can be broadly defined as youth who are medically, socially or economically vulnerable. Managing the medical issues of YSHCN in the context of their developmental needs may present a challenge for clinicians. Perhaps this is why common consultations with YSHCN often involve issues that extend beyond disease management into areas such as medical non-adherence, relationships, mental health, sexuality, and psychosocial concerns. Effective consultations of YSHCN therefore require more than a knowledge of disease, as clinicians must engage the youth and inquire more broadly to address issues comprehensively.

In this paper we will review factors critical to youth consultation with a focus on YSHCN. Additionally, we will introduce a novel clinical approach which we refer to as the coaching stance. We will also introduce the framework TGROW, which is derived from the coaching literature, and provide an example of its application using a clinical scenario [4].

## 2. Back to Basics

When consulting with YSHCN, it is critical to get back to basics by considering typical adolescent development. The period of adolescence and young adulthood between the ages of 12–24 is marked by myriad asynchronous biopsychosocial changes that are thoroughly reviewed in *Adolescent and Young Adult Healthcare: A practical guide. 6th ed* [5]. Youth are developing abstract thinking, focusing increasingly on body and appearance and are experimenting with new behaviours; all interconnected aspects of the journey towards independence. Adolescents naturally shift to adopt peer codes and lifestyles while establishing sexual, ego, vocational and moral identities [5,6].

In their clinical encounters many youth desire autonomy, a non-judgmental attitude, and a safe space to make decisions [7]. Youth should be given the opportunity to be seen alone and assured of confidentiality. Establishing boundaries around confidentiality as an initial aspect of engagement affords the opportunity to clarify when the need for intervention precludes the need for privacy. It is equally important to inquire about ground rules that go beyond the tenants of confidentiality; allow the youth to express what is important for them in the clinical relationship and who they may wish to involve in their healthcare [8]. For comprehensive care, practitioners should use standardized screening tools such as HEADSS [9], or the newer pneumonic framework SSHADESS, which emphasizes strengths, see Box 1 ([10,11], Appendix A).

Box 1 **The SSHADESS Screen.**

**S**trength

**S**chool

**H**ome

**A**ctivities

**D**rug/Substance Use

**E**motions/Eating/Depression

**S**uicidality

**S**afety

Comprehensive adolescent assessment tools and resources are available and include the American Academy of Pediatrics’s Bright Futures guidelines, the American Medical Association’s Department of Adolescent Health Guidelines for Adolescent Preventive Services, and the Canadian Paediatric Society’s Greig Health Record [12,13,14].

## 3. Youth with Special Health Care Needs

In addition to adapting to life with a chronic illness, YSHCN must also traverse the same stages of psychosocial development as their peers [2,5,6]. It is not surprising then, that when compared to their same-age peers, adolescents with chronic illness are at higher risk of developing emotional and social issues, and are at least as likely to engage in concerning risk-taking behaviours [15].

There are many ways that chronic illness can impact adolescent development. The path of physical and psychosocial development in YSHCN can be altered by a specific disease state, as well as the developmental age at which the illness was first diagnosed [16]. Risk-taking behaviours which impact medical management or the disease process can have severe and long-lasting ramifications [15]. Chronic illness can also radically impact the development of identity by altering appearance and by creating physical limitations [17]. Medical matters can create the requirement for ongoing care and vigilance, interrupting adolescent experience and precluding future planning in multiple domains. Existential questions can emerge from uncertainties about disease progression and life expectancy.

As healthcare providers, we must be equipped to meet the comprehensive issues of YSHCN. A position statement from the Canadian Paediatric Society speaks to how healthcare providers can support YSHCN and their caregivers by considering interactions between developmental milestones and specific disease management challenges [18]. The statement highlights important areas for clinician exploration including independence and separation, decision-making and adherence, self-care and disease management, and risk-taking behaviours. In a 2006 paper on the role of adolescent development in the transition process, Kaufman states that adolescents with transplants need to have the developmental changes in areas of cognition, autonomy, sexuality, peer relevance, morality and philosophy be both recognized and factored into models of care [6]. An awareness of these universal developmental needs should inform the attitude and approach of the consultant working with all YSHCN, ranging from cystic fibrosis to bipolar disorder.

## 4. Considerations for Care

Youth are interested in HCP who emulate honesty, experience, and knowledge [19]. Recent literature reviews have highlighted that conceptual frameworks consistent with strength-based approaches, such as resilience and positive youth development, bolster clinical problem solving, empower youth to become increasingly self-aware and foster self-management [20]. Consultants working with YSHCN must be aware of the medical implications of the disease state, and also need to be invested in an approach that will promote trust and accountability in the clinical relationship.

The clinical management of YSHCN, either as a consultant or primary provider, presents challenges which can strain the relationship between youth and HCP. Patients and HCP may have different goals and priorities. It may also be difficult for HCP to choose whether to focus on medical or on developmental needs. Medical acuity may amplify these challenges and compel the HCP to assume a rigid stance, thereby imposing a hierarchy which may impact the relationship negatively.

Risk-taking in the form of illness mismanagement is a common example of the types of challenges experienced in these clinical relationships. Although risk-taking in adolescents is normal and can in fact signal healthy development, HCP may feel compelled to respond harshly to prevent negative health outcomes. Alternately, there may be questions of how best to support a youth who is falling short of assuming independent care of illness. Although the HCP is aware of their role in fostering appropriate developmental skills including autonomy, they may be understandably reluctant to risk adapting their level of support to foster this outcome.

When faced with these dilemmas, HCP may default to a more traditional prescriptive stance which relies mostly on medical expertise. Paradoxically, when physicians engage in this manner, the adolescent may be less likely to engage with trust, thereby precluding discussions which could foster patient well-being in many domains including enhanced self-directed health behaviours.

## 5. Beyond the Basics: What Else?

In addition to the traditional medical approach, we propose the adoption of a complimentary stance that we refer to here as the coaching stance (see Table 1). The coaching stance describes an interpersonal style which shares common elements with other therapeutic and counselling interventions, including motivational interviewing. The coaching stance is not intended to act as a substitute for motivational interviewing or for other established psychotherapies. The coaching stance consists of five factors: non-judgment; curiosity; empathy; openness, and; flexibility. The coaching stance invites the provider to engage with authenticity and does not require specific training or expertise in any particular therapeutic modality. Rather, adopting the coaching stance allows the HCP to broaden their understanding of how they can engage as a helper, utilizing the interpersonal skills they already possess. Clinicians can fully embrace the spirit of collaboration with the patient who is ultimately in charge of decision-making whenever possible and appropriate. The deferral to a prescriptive dynamic can be reserved for circumstances that are acute and life-threatening where the assumption of physician control of the relationship is imperative. The medical stance and the coaching stance are not in opposition to each other, rather, they are complimentary with each having its relevance and utility. The need to shift between these two stances may create tension for patient and provider, and highlights the need for explicitly defining the tenants of the clinical relationship at the outset.

Integrating both the medical stance and the coaching stance into clinical consultations expands possibilities for how HCP can support YSHCN (see Table 2). This stance contributes to a trusting, mutually respectful relationship where honest disclosure and a full exploration of issues is more likely to occur; in both the initial consultation and over time. When employing the five factors, the form of questioning examines the “how” and “what” of the issues (i.e., “How might you do this differently?”), and focuses less on the “why”, which can be experienced as judgmental or directive (i.e., “why did you do that!?”). Topics are explored more broadly and extensively with phrases like “Tell me more” and “what else?” Using this approach, it is also advantageous to incorporate a coaching tool called “Naming it.” Naming it brings to light hidden agendas or issues that are challenging to talk about but are sensed by the HCP. This is where one is not only listening with intention, but also using intuitive skills to pick up on body language and changes in the energy of the adolescent. For example, the HCP may sense a youth is not taking all their prescribed medication because the youth hesitates and looks away when answering a question about frequency of emergency department visits. When naming it, the HCP would say “I want to help you understand your increasing need for emergency visits, maybe it has something to do with your medication routine?”

## 6. TGROW

The following case example demonstrates the application of the coaching stance utilizing a coaching framework known as TGROW (see Box 2). TGROW is an approach to identifying priorities and generating solutions which holds promise in clinical and in non-clinical settings. Further reading on the topic of coaching for those interested in integrating it into their practice can be found in the book *Effective Modern Coaching: The Principles and Art of Successful Business Coaching* by Myles Downey. This book is written specifically for business coaching, however, the concepts can be easily applied to the healthcare setting [4].

The following example involves on a non-urgent clinical encounter where the coaching stance is optimally employed. We can assume that confidentiality and ground rules were established in this scenario, and that a physical exam and medical history including the use of a screening tool has already been completed.

Box 2 **TGROW: A Structured Approach to Problem Solving.**

**Steps****Intended Outcome****T:** TOPICEstablish what the patient wants to talk about.**G:** GOALDecide what will be achieved by the end of the encounter.**R:** REALITYGenerate the clearest possible picture of the situation.**O:** OPTIONSEstablish a sense of all the possibilities for achieving the goal.**W:** WRAP-UPSelect the most appropriate option and agree on next steps.

## 7. Case Example and Discussion of the Coaching Stance and TGROW Approach

### 7.1. Clinical Vignette

Box 3 below “The coaching Stance and TGROW in action” illustrates the case of MB is a 15-year-old female with insulin-dependent diabetes mellitus (IDDM), diagnosed at age 10. She lives with her father, step-mother and their six-year-old son. She is a grade 10 student with interests in music. She has been referred by her endocrinologist because of poor insulin control and a suspicion of binging and purging behaviour through the omission of insulin. She has had two brief admissions in the last 24 months for Diabetic Ketoacidosis.

In consultation with you, her parents express exasperation. They have had multiple family meetings with the endocrinologist and with the team social worker to figure out how to help their daughter improve her illness management. “We can’t get her to do this, we’ve tried everything. She needs to realize how important this is. She’s old enough to know better and we are so tired of this behaviour. She could kill herself and she doesn’t seem to care. We monitor her every day and even that’s a fight—she doesn’t want us to see her numbers and we know she lies to us about all of this. We can’t move on as a family until this is fixed!”

When seen individually, MB is polite and appropriate. She states that she does not know why her A1C is so high, and minimizes the concerns around binging and purging, stating that she has “barely ever done that”. She explains that she does maintain her IDDM “for the most part”. She is also tired of everyone talking about it all the time and “trying to scare her”. She hates coming to these appointments.

Box 3 **“The Coaching Stance and TGROW in action”**

HCP: “I have a referral from your endocrinologist and have read about their concerns. I’m curious; what do you think would be most important for us to talk about today?”

MB: “I don’t know. I’m so sick of talking about my diabetes. Seriously, it’s all anyone ever talks about.”

HCP: “Oh, being sick of diabetes talk sounds like an important topic; could that be?”

MB: “Sure.”

HCP: “OK. Well, if we’re talking about how sick you are of the diabetes, what do you think would be helpful to achieve…like what would be good for us to get to by the end of the session?”

MB: “Well, I don’t know. I have diabetes and it sucks. I don’t see that changing.”

HCP: “I see your point. You mentioned that you were sick of everyone talking about it—could there be a way to change that? Somehow?”

MB: “Well, that would be good…but I doubt it could happen”

HCP: “So the goal might be to find some way…even a small way…for you to have to talk less about diabetes? Did I get that right?”

MB: “OK.”

HCP: “Tell me about this issue, this ‘talking about diabetes’…I really want to hear about it.”

MB: “My parents are constantly talking about it; they don’t want to talk about anything else. Unless I show them my book and number they don’t seem like they care about anything. If my numbers aren’t good they basically get mad or disappointed and I get another lecture. My stepmother basically hates me and has stopped talking to me because she thinks I’m irresponsible and selfish because I don’t manage my diabetes all the time. Same thing at the doctor; they keep telling me I’m going to go blind or lose my legs. Honestly, I used to care when they said stuff like that, but I don’t care anymore.”

HCP: “That does sound exhausting. I can see why you’re sick of talking about it—it seems like it can be really negative.”

MB: “Oh, it’s never good enough for them.”

HCP: “So what I hear you say is that you don’t get to talk to your parents about other things because your diabetes care is always the number one topic…is that right?”

MB: “Yes. They are totally obsessed with it…it’s not their life!”

HCP: “Fair enough, it’s your life.”

MB: “Exactly!”

HCP: “Is there anything else that would help me understand why you’re so sick of talking about diabetes?”

MB: “No, I think that’s basically it.”

HCP: “OK. I’m wondering now what would have to change for things to be different?”

MB: “They would have to stop worrying and get over it.”

HCP: “Your parents?”

MB: “Everyone.”

HCP: “What if that doesn’t happen? What else could change?”

MB: “Um…maybe if they just stopped talking to me in general.”

HCP: “Oh…is that something you would want?”

MB: “No. I want to work on my relationships…especially with my stepmother. We used to be close before the DM.”

HCP: “Oh…so before DM became what everyone talked about, you used to have a good relationship?”

MB: “Yes. We did all kinds of stuff together—it was fun. But now she’s just mad at me all the time.”

HCP: “What would you be talking to her about if it wasn’t about the DM?”

MB: “I’m not sure…we used to go to shows together…we both like musical theatre…maybe I should start talking to her about that stuff again.”

HCP: “What could happen if you did?”

MB: “I don’t know. She might not want to.”

HCP: “How could you check that out?”

MB: “I guess I could ask her.”

HCP: “Ask her what?”

MB: “Maybe ask her if she wants to hang out and talk about musicals?”

HCP: “Anything else?”

MB: “Maybe I could ask her to not talk about the diabetes?”

HCP: “What do you think of that idea? To ask her if she wants to hang out and talk about musicals and NOT diabetes?”

MB: “I don’t know if she’d go for it…I’m pretty sure I’d still have to show her my book.”

HCP: “OK. Would you still meet your goal of not talking about DM so much…even if you showed her your book?”

MB: “Yes. I still wish I didn’t have to show her the damn book.”

HCP: “I understand. So will you be following up with this plan?”

MB: “Yes, I can talk to her.”

HCP: “When?”

MB: “Today.”

HCP: “Is there any way I can help you to follow through with this idea? I’m available if you want to connect with me. Let me know how it goes! I’ll be curious to see what happens.”

### 7.2. Topic

In this case, if guided only by disease concerns and the emotional tone of the parents, one would easily have assumed that the topics to be addressed were diabetes non-compliance and eating disorder concerns. Using the coaching stance, we refrain from making such assumptions and are curious about the topic the young person feels is most relevant.

Here, the physician clearly identified that they are aware of the issues related to disease management and also family stressors. What the physician did not do is doggedly pursue these topics, but rather focused on youth engagement by being non-judgmental and curious. This approach allowed the patient to reflect on what is most important for them, signalling that the physician respects their autonomy. It further allowed the youth to practice skills of reflective thinking and communication.

The concerns prompting the referral are of critical importance and require future inquiry, but they do not present an acute risk. Employing the coaching stance initially, enables future conversations on these topics to be rooted in a relationship of respect and trust which will likely yield more honest disclosure.

### 7.3. Goal

When setting a goal for a clinical encounter, one attempts to establish an outcome that can be achieved by the end of the encounter. Meeting a patient’s goal for the session can go a long way to establishing trust. Finding “a small way of getting people to talk less about DM” was achievable here and opened the door to rich discussion about the patient’s experience of living with illness. By being open to the patient’s experience, and flexible in the clinical encounter, the clinician successfully invited the patient to select a goal. Thereafter the hierarchical dynamic that often characterizes clinical encounters was allowed to shift; clinician and patient could work together on achieving the goal.

### 7.4. Reality

When pursuing the patient’s realities one is coming in without assumptions and is allowing the youth to tell their story. The facts will be told from the youth’s perspective, which is critical as it is the youth with whom you are partnering to achieve the goal. Rather than correcting facts or questioning assumptions, expressing empathy demonstrates interest in their experience and encourages more disclosure. In this case, the youth was able to express exactly what was going on that she disliked, which created the opportunity to generate options for change.

### 7.5. Options

In seeking options, one attempts to support the individual to think broadly about what possibilities exist towards the achievement of their stated goal. By remaining open to all options the youth is able to discuss possible solutions and evaluate them with support. Creativity and reasoning skills are practiced by allowing the youth to verbalize and consider all the possible solutions.

### 7.6. Wrap up

Ultimately, there needs to be a decision about which options to choose and a sense of what to do next. Ideally, there will be a commitment to some action in service of the goal. However, this is not essential. Perhaps the decision requires further reflection or exploration. Fundamentally, one is supporting the youth to make decisions and to take autonomous steps while offering our assistance should they wish to have it.

## 8. Conclusions

Issues faced by YSHCN compel the HCP to move beyond the basics of standard care and to ask themselves, “what else can I do to meet the comprehensive needs of this population?” In this paper we have outlined the basics of adolescent development and assessment. We have highlighted special considerations for the care of YSHCN, discussing some of the complexities that might require consultants to adopt a broader approach. There are limitations to this approach and it may not be effective with all youth. Notably, youth with significant mental health or cognitive challenges may require the additional expertise of those that specialize in more established therapeutic models. Some youth will require the expertise of specialized mental healthcare practitioners. Consultants may consider incorporating the coaching stance and TGROW, novel in the field of healthcare, into their practice repertoire, as both are complimentary to a more traditional medical approach. Practitioners may initially be required to invest more time to establish the clinical relationship, however, possible clinical implications of this approach may include improved disease management, and enhanced psychosocial development. Future directions might include investigations of coaching tools and techniques in clinical settings, specifically with the adolescent population with chronic illness.

## Figures and Tables

**Table 1:** Coaching Stance: The Five Factors.

**Non-Judgment**	“If there is no right or wrong explanation, how would you describe this situation to your best friend?”
**Curiosity**	“How do you think things are going?”
**Empathy**	“It seems like a difficult situation.”
**Openness**	“What other factors do you think we should consider?”
**Flexibility**	“What else do you think is important here?”

**Table 2:** Comparison of Medical and Coaching Stances.

Stance	Relationship Style	Clinical Goal Definition	Primary HCP Methods
**Medical**	Hierarchical/ Patriarchal	Often assumed Defined by medical expertise and physician priorities	Telling—“This is what you should know/do!” Providing medical information Reinforcing compliance by engaging parents and by the heightening of urgency (scare tactics) Use of narrow specific questions: (i.e., Why aren’t you using your medication as prescribed?)
**Coaching**	Collaborative/ Empowering	Discovered collaboratively, patient autonomy is prioritized (dependent on developmental age and medical acuity)	Asking—“Tell me more?” Listening Supporting autonomous goal attainment using the five factors: Non-judgment Curiosity Empathy Openness Flexibility HOW and WHAT questions Broad questions: “What else might you think of to solve this problem?” Naming it

## Appendix A

**The SSHADESS Screen**	
**Key Questions** you might ask in a SSHADESS (Strength, School, Home, Activities, Drug/Substance Use, Emotions/Eating/Depression, Suicidality, Safety) screen follow. Positive or concerning responses will require a deeper level of questioning than included here.	
**Strengths**: What do you like doing? How would you describe yourself? Tell me what you’re most proud of. How would your best friends describe you?	**Drugs/Substance Use**: Do any of your friends talk about smoking cigarettes, taking drugs, or drinking alcohol? Do you smoke cigarettes? Drink alcohol? Have you tried sniffing glue, smoking weed, or using pills or other drugs? When/if you smoke, drink, or get high, how does it make you feel, or what does it do for you?
**School**: What do you enjoy most/least about school? How many days have you missed or had to be excused early or arrived late to school? How are your grades? Any different from last year? Do you feel like you are doing your best at school? (If no) Why not? What’s getting in the way? Do you feel safe on the way to school and in school? Do you participate in gym class? What would you like to do when you get older?	**Emotions/Eating/Depression**: Have you been feeling stressed? Do people get on your nerves more than they used to? Are you feeling more bored than usual? Do you feel nervous a lot? Have you been having trouble sleeping lately? (If yes) What kind of trouble? Would you describe yourself as a healthy eater? Have you been trying to gain or lose weight? Tell me why. Have you been feeling down, sad, or depressed? Have you thought of hurting yourself or someone else? Have you ever tried to hurt yourself?
**Home**: Who do you live with? Any changes in your family? Could you talk to your family if you were stressed? Who would you go to first?	**Sexuality**: Are you attracted to anyone? Tell about that person. (Using gender-neutral language) Are you comfortable with you sexual feelings? Are you attracted to guys, girls, or both? What kind of things have you done sexually? Kissing? Touching? Oral sex? Have you ever had sexual intercourse? Have you enjoyed it? What kind of steps do you take to protect yourself? Have you ever been worried that you could be pregnant? Have ever been worried about or had a sexually transmitted infection?
**Activities**: Are your friends treating you well? Do you have a best friend or adult you can trust outside your family? Are you still involved in the activities you were doing last year? What kind of things do you do just for fun? Are you spending as much time with your friends as you used to?	**Safety**: Are there lots of fights at your school? Do you feel safe at school? Is there bullying? Have you been bullied? Do you carry weapons? What kinds of things make you mad enough to fight? Has anyone every touched you physically or sexually when you didn’t want them to? Does your boyfriend/girlfriend get jealous? (Jealousy is an early sign of controlling, potentially abusive, relationships.) Do you ever get into fights with your boyfriend/girlfriend? Physical fights? Have you ever seen people in your family or home hurt each other? Say mean things? Throw things or hit each other?
